# In vivo detection of tau fibrils and amyloid β aggregates with luminescent conjugated oligothiophenes and multiphoton microscopy

**DOI:** 10.1186/s40478-019-0832-1

**Published:** 2019-11-08

**Authors:** Maria Calvo-Rodriguez, Steven S. Hou, Austin C. Snyder, Simon Dujardin, Hamid Shirani, K. Peter R. Nilsson, Brian J. Bacskai

**Affiliations:** 1 Alzheimer Research Unit, Department of Neurology, Massachusetts General Hospital and Harvard Medical School, 114, 16th St, Charlestown, MA 02129 USA; 2 Department of Physics, Chemistry and Biology, Linköping University, 581 83 Linköping, Sweden

**Keywords:** Alzheimer’s disease, Amyloid beta plaques, Cerebral amyloid angiopathy, Luminescence, Multiphoton microscopy, Neurofibrillary tangles, Oligothiophenes, Tau

## Abstract

The detection of amyloid beta deposits and neurofibrillary tangles, both hallmarks of Alzheimer’s disease (AD), is key to understanding the mechanisms underlying these pathologies. Luminescent conjugated oligothiophenes (LCOs) enable fluorescence imaging of these protein aggregates. Using LCOs and multiphoton microscopy, individual tangles and amyloid beta deposits were labeled in vivo and imaged longitudinally in a mouse model of tauopathy and cerebral amyloidosis, respectively. Importantly, LCO HS-84, whose emission falls in the green region of the spectrum, allowed for the first time longitudinal imaging of tangle dynamics following a single intravenous injection. In addition, LCO HS-169, whose emission falls in the red region of the spectrum, successfully labeled amyloid beta deposits, allowing multiplexing with other reporters whose emission falls in the green region of the spectrum. In conclusion, this method can provide a new approach for longitudinal in vivo imaging using multiphoton microscopy of AD pathologies as well as other neurodegenerative diseases associated with protein aggregation in mouse models.

## Introduction

With more than 30 million people worldwide suffering from dementia, Alzheimer’s disease (AD) is the most common age related form. Despite the efforts of the research community, the underlying mechanisms are still unknown, and as of now there is no cure. The pathological hallmarks of AD are the presence of extracellular plaques, composed of aggregated amyloid β (Aβ) peptides, and intracellular neurofibrillary tangles (NFTs), composed of tau proteins hyper and abnormally phosphorylated. The aberrant accumulation of Aβ plaques and NFTs has been implicated as a critical event in the pathology of AD, and precedes cognitive decline [1]. The in vivo detection of these two hallmarks enables investigators to investigate critical questions and further understand the etiology of the disease.

Luminescent conjugated oligothiophenes (LCOs) have been developed as a promising tool for fluorescence imaging of protein aggregates [2, 3]. They present electronically delocalized conjugated thiophene backbones, which gives them specific intrinsic fluorescence characteristics and allows for different detection modes based on their fluorescence excitation/emission spectra and fluorescence lifetimes [4]. Recently, a full palette of LCOs showing different excitation/emission wavelengths have been synthesized, allowing multiplexed detection methodologies by combining LCO variants with different dyes or fluorophore-labelled antibodies targeting different proteins [3]. LCOs have been used previously for ex-vivo staining on human and mouse brain tissue by fluorescence microscopy and have been found to label NFTs (i.e. fibrillary tau, but no soluble tau, [5]), and Aβ plaques and cerebral amyloid angiopathy (CAA) at high contrast and specificity [6]. Importantly, they offer the ability of crossing the blood brain barrier (BBB) to stain NFTs and Aβ pathology in vivo [5]. Due to their specificity and pronounced enhancement of the emission intensity upon interaction with protein aggregates, we tested whether they could be used for labelling AD pathology (NFTs, Aβ plaques and CAA) in vivo in different mouse models, and if they could be imaged in the living mouse brain with multiphoton microscopy after implanting a cranial window. Two LCO variants, HS-84 (single photon excitation maximum is ~ 430 nm, emission maximum ~ 512, 547 nm), and HS-169 (excitation maximum is ~ 375 nm and ~ 535 nm (double excitation peaks), emission maximum ~ 665 nm) [3], were used to label NFTs and amyloid pathology in vivo.

## Methods

### Animals

Mouse experiments were performed with the approval of the Massachusetts General Hospital Animal Care and in compliance with the National Institutes of Health guidelines for the use of experimental animals. Mice used included: 1) APPswe:PSEN1∆E9 double transgenic (Tg) mice (APP:PS1) (The Jackson laboratory, B6.Cg-Tg (APPswe, PSEN1dE9)85Dbo/Mmjax), expressing both the human *APP* gene carrying the Swedish mutation K594n/M595 L and the exon 9 deletion mutation in the *PS1* gene. In this model, amyloid pathology (Aβ plaques and CAA) start to deposit around 5-months of age [7]. Six- to 11-month-old APP:PS1 Tg mice of either sex, along with age-matched Wt littermate controls were used; 2) 8–11 month-old rTg4510 Tg mice of either sex (The Jackson laboratory, Tg (Camk2a-tTA)1Mmay Fgf14Tg (tetO-MAPT*P301L)4510Kh strain that expresses the tetracycline-controlled transactivator protein (tTA) [8]), along with age-matched Wt littermate controls were used, and 3) APP:PS1-rTg4510 (FVBB6F1rTg4510(App/PSEN1)85) (APP:PS1-rTg4510 mice), which exhibits mixed pathology of amyloid β plaques and NFTs [9]. Mice were socially housed at 3–4 animals per cage with ad libitum access to food and water on a 12/12 h light/dark cycle with controlled conditions of temperature and humidity.

### Cranial window implantation and LCO delivery

Cranial window surgery was performed as previously described [10] with minor modifications. Mice were anesthetized with 1.5% (vol/vol) isoflurane and placed in a stereotactic apparatus. A piece of skull (measuring 3 mm in diameter) over the left somatosensory cortex was removed, replaced with a 5 mm diameter glass coverslip and fixed with a mixture of Krazy Glue and dental cement [11]. Body temperature was maintained at 37C throughout the full procedure by using a heated pad. Mice were given buprenorphine (0.1 mg/kg) for 3 days following surgery, and were allowed to recover for at least 3 weeks before they were imaged.

HS-84 and HS-169 were synthesized as described previously [3] and diluted with PBS to a stock concentration of 5 mg/mL. To label amyloid pathology and NFTs in vivo, 150 nmol of LCO in 150 μl PBS [3] was intravenously delivered via retro-orbital injection [12]. A subset of APP:PS1 Tg mice were injected IP with Methoxy-X04 (363 nmol in 280 μl PBS) 24 h before the imaging session to produce high contrast images of Aβ plaques (emission 460–500 nm) [13].

To label NFTs in vivo with Thiazine Red in rTg4510 mice [14], dura matter was removed and 0.5 mg/ml Thiazine Red in PBS was topically applied onto the brain for 1 h, and then thoroughly washed with PBS. An 8 mm cranial window was implanted and sealed with a mixture of Krazy Glue and dental cement. HS-84 was injected 1 week before the experiment.

### In vivo multiphoton imaging and data analysis

In vivo imaging was performed on anesthetized mice (1.5% isoflurane). Images of amyloid pathology, NFTs and dextran angiograms were obtained using an Olympus FluoView FV1000MPE multiphoton laser-scanning system mounted on an Olympus Bx61WI microscope and an Olympus 25x dipping objective (NA = 1.05). A Deep-See Mai Tai Ti:Sapphire mode-locked laser (Mai Tai; Spectra-physics) generated two-photon excitation at 800 nm, and three photomultiplier tubes (PMTs) (Hamamatsu) collected emitted light in the range of 380–480, 500–540 and 560–650 nm [15]. Settings and laser power remained unchanged throughout the different imaging sessions. Either Texas Red dextran or fluorescein dextran (70,000 Da MW; 12.5 mg/mL in PBS; Molecular Probes) was retro-orbitally injected before every imaging session to provide a fluorescent angiogram. The brain was imaged at depths of up to 200 μm from the surface of the brain. Three to eight cortical volumes (Z-series, 127 μm × 127 μm, 200–300 μm depth) were acquired per mouse, at a resolution of 512 × 512 pixels. Imaged volumes were randomly chosen. Images were exported and processed using the Fiji package of ImageJ (National Institutes of Health). Fluorescence intensity of each Aβ plaque, NFT or individual blood vessel was quantified using the ImageJ measure tool. Images presented in the figures are either single slices or maximum intensity image projections of the 3D volumes.

### Ex-vivo staining and immunohistochemistry

At the end of the last imaging session, mice were euthanized under CO_2_, perfused with PBS, and brains were removed, flash frozen and sliced into 20 μm coronal sections on a cryostat (Leica). To validate labelling by HS-84 and HS-169, slices were exposed to either 0.005% Thioflavin S (ThioS) or 0.005% Thiazine Red in EtOH for 5 min and then washed with 80% EtOH followed by TBS. Slices were mounted with DAPI Vectashield and subjected to confocal imaging to address the colocalization of either HS-84/Thiazine Red or HS-169/ThioS. Additionally, sections were subjected to heat induced epitope retrieval in 10 mM citric acid, 0.05% Tween 20, pH 6.0, permeabilized with 0.5% triton X-100 and incubated with anti-tau antibodies against Alz50 (kindly provided by Dr. Peter Davies [16], 1:100) or 6E10 (anti-β-amyloid, 1–16 antibody, Biolegend, 1:100) overnight at 4 C. Appropriate secondary antibodies (goat anti-mouse IgM heavy chain secondary antibody, Alexa Fluor 568 conjugate or Alexa Fluor 647 conjugate respectively, Invitrogen, 1:400) were applied and incubated for 1 h at room temperature. Slices were mounted with DAPI Vectashield (Vector Laboratories) and subjected to fluorescence imaging. Images were recorded using the VS120 Virtual Slide Microscope system (Olympus). Ex vivo staining/co-staining with both LCOs was carried out in APP:PS1 and APP:PS1-rTg4510 free floating sections. Sections were stained for 5 min in 0.005% (w/v) HS-84 and/or HS-169 prepared in 50% EtOH in TBS, and then washed with 80% EtOH followed by TBS. Slices were mounted with Vectashield and subjected to confocal imaging.

### Statistical analyses

GraphPad Prism 6 was used for statistical analyses. The intensity changes of HS-84 and HS-169 over time were analyzed using the Friedman test (one-way ANOVA with repeated measures). Significance levels were set at *p* < 0.05. Data are presented as mean ± SEM.

## Results

### HS-84 binds to NFTs and can be detected with multiphoton microscopy

**Fig. 1:**
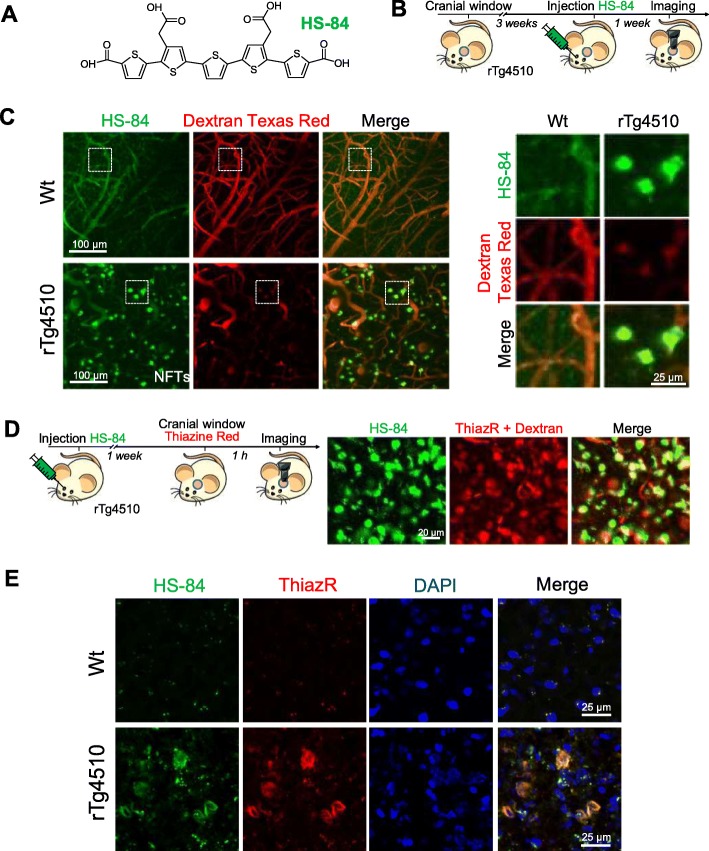
HS-84 binds to NFTs in the rTg4510 Tg mouse and can be detected with multiphoton microscopy*.* **a**. HS-84 molecule. **b**. Experimental procedure to characterize HS-84 in the mouse brain in vivo*.* rTg4510 Tg mice and Wt littermates carrying a cranial window were injected with HS-84 via retro-orbital and subjected to intravital multiphoton microscopy. **c**. In vivo multiphoton microscopy images of HS-84 in Wt (top) and rTg4510 Tg mouse (bottom). Pictures show HS-84 (green), Dextran Texas Red (70,000 Da MW, red), and merge of both channels. Insets are shown on the right. Representative from *n* = 3 Wt and *n* = 6 rTg4510 Tg mice. **d.** In vivo validation of HS-84 labelling NFT. Left, experimental procedure to validate HS-84 in the mouse brain in vivo*.* rTg4510 Tg mice were injected with HS-84. 1 week later, Thiazine Red was directly applied for 1 h onto the brain after skull and dura removal. A 8 mm cranial window was implanted and the mouse was subjected to multiphoton microscopy. Right, HS-84 colocalizes with Thiazine Red. Representative of *n* = 2 rTg4510 Tg mice. Scale bar represents 20 μm. **e**. Post-mortem validation of NFTs labelled with HS-84 in the mouse brain. HS-84 was injected via retro-orbital and the mice were euthanized 1 week later. Brains were sliced in a cryostat and incubated with 0.005% Thiazine Red in EtOH. Colocalization of both dyes can be appreciated. Scale bar represents 25 μm and applies to all pictures

**Fig. 2:**
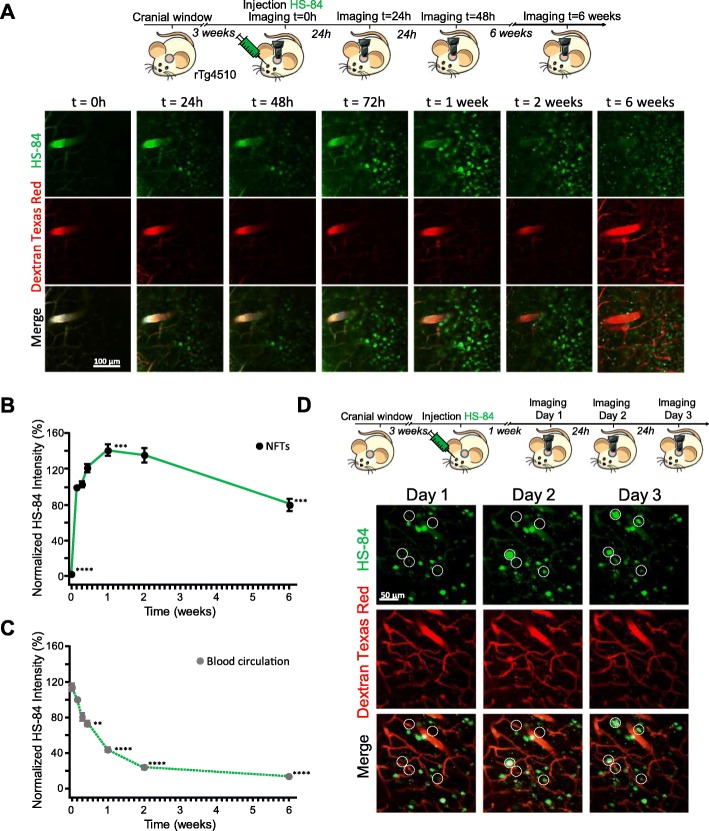
Optimal visualization window for HS-84 with multiphoton microscopy in the rTg4510 mouse brain in vivo. **a**. Top panel, experimental procedure to characterize HS-84 in the mouse brain in vivo*.* rTg4510 Tg mice were retro-orbitally injected with HS-84 and longitudinally imaged with multiphoton microscopy for 6 weeks. Bottom panel, pictures show different time points after injection of HS-84 (green). T = 0 represents imaging few minutes after injection. Dextran Texas Red 70,000 Da MW (red) was systematically injected to create a fluorescent angiogram. Scale bar represents 100 μm and applies to all pictures. **b**. Quantitative analysis of fluorescence intensity of HS-84 binding to NFTs. Normalized to t = 24 h. Optimal imaging time was determined to be 1 week after HS-84 injection in the rTg4510 Tg mouse. *n* = 111 cells from 2 mice. **c**. Quantitative analysis of fluorescence intensity HS-84 in the blood circulation. Normalized to t = 24 h. *n* = 48 vessels from 2 mice. **d**. Dynamics of NFTs formation in the rTg4510 Tg mouse. Top panel, experimental procedure to characterize formation of NFTs in the mouse brain in vivo*.* rTg4510 Tg mouse with a window already implanted was intravenously (retro-orbital) injected with HS-84 and longitudinally imaged with multiphoton microscopy for 3 consecutive days. Bottom, pictures show 3 successive days 1 week after injection of HS-84 (green). Dextran Texas Red 70,000 Da MW (red) was systematically injected. Scale bar represents 50 μm and applies to all pictures. New NFTs can be observed within 1 day

First, we addressed whether the delivery of HS-84 (Fig. 1a) to the mouse brain would specifically label NFTs and whether its fluorescence could be detected with multiphoton microscopy. rTg4510 Tg mice were used as a model of tauopathy. This mouse model expresses mutant human tau (P301L mutation) directed to the forebrain, and develops NFT pathology in the cortex, resembling the human AD type of tauopathy [8]. Wt littermates were used as control. HS-84 was injected intravenously (retro-orbitally) 1 week prior to the imaging session in mice with a cranial window already implanted (Fig. 1b). Dextran Texas Red (70,000 Da MW) was systematically injected to create a fluorescent angiogram [17]. HS-84 was excited at 800 nm. Figure 1c shows representative images of NFTs selectively labeled with HS-84. No significant fluorescence could be detected in the Wt mice. To validate that HS-84 was labelling NFTs in the mouse brain in vivo, we co-labelled with Thiazine Red, a dye previously described to selectively stain NFTs [18], in the living brain. rTg4510 Tg mice previously injected with HS-84 were subjected to craniotomy and dura removal. Thiazine Red (0.5 mg/ml) was directly applied onto the brain and then the mouse was imaged with multiphoton microscopy. Thiazine Red was topically applied to the brain since it was found not to cross the BBB. Colocalization of both dyes supports the fact that HS-84 stains NFTs and that it can be imaged with intravital multiphoton microscopy (Fig. 1d). To further validate HS-84 labelling NFTs in the mouse brain, post-mortem staining with Thiazine Red and an antibody directed against a pathological form of tau (Alz50) was performed to probe colocalization (Fig. 1e and Additional file 1: Figure S1). To determine the optimal time window of imaging after HS-84 injection, we next performed longitudinal multiphoton imaging of the same volumes in the same mouse at 7 different time points within a 6 week period. rTg4510 mice with a cranial window previously implanted were injected with 150 nmol HS-84 and imaged at t = 0 (right after injection), and later times (Fig. 2a). The optimal imaging time (time with strongest fluorescent labelling) was determined to be 1 week after HS-84 injection in the rTg4510 Tg mouse (Fig. 2b). The calculated fluorescence intensity did not include the blood vessels. It was observed that a single injection was enough to follow the formation of new NFTs, since HS-84 remained in the circulation (Fig. 2c), and stained newly formed NFTs for at least 3 consecutive days (Fig. 2d), which were labeled within 24 h in this mouse model. We also attempted to image NFTs labelled with the LCO HS-169 following the same procedure. No cellular fluorescence could be detected with multiphoton microscopy after intravenous injection (Additional file 2: Figure S2), suggesting the lack of (or weak) in vivo binding of HS-169 to NFTs, although it has been reported that HS-169 labels NFTs ex-vivo [3]. To test the hypothesis of a weaker labeling of HS-169 to the NFTs, we stained tissue sections from APP:PS1-rTg4510 Tg mice [9], which exhibit both plaques and tangles. Staining was performed with either HS-84 or HS-169 at same concentration, washing times and settings in the microscope. We could observe that both LCOs bound easier to amyloid β plaques and therefore the fluorescence obtained from plaques was more intense (Additional file 2: Figure S2C). On the other hand, we needed to increase laser % and gain in order to observe NFTs, and even more when labeled with HS-169 (notice and increased background in Additional file 2: Figure S2C, saturated, right). This suggests that likely the concentration of HS-169 injected in vivo was not enough to observe the NFTs under two photon microscopy, whereas it was sufficient for HS-84. In order to avoid toxicity, we decided to not inject the LCO more concentrated.

### HS-169 binds to amyloid β plaques and CAA and can be detected with multiphoton microscopy

**Fig. 3:**
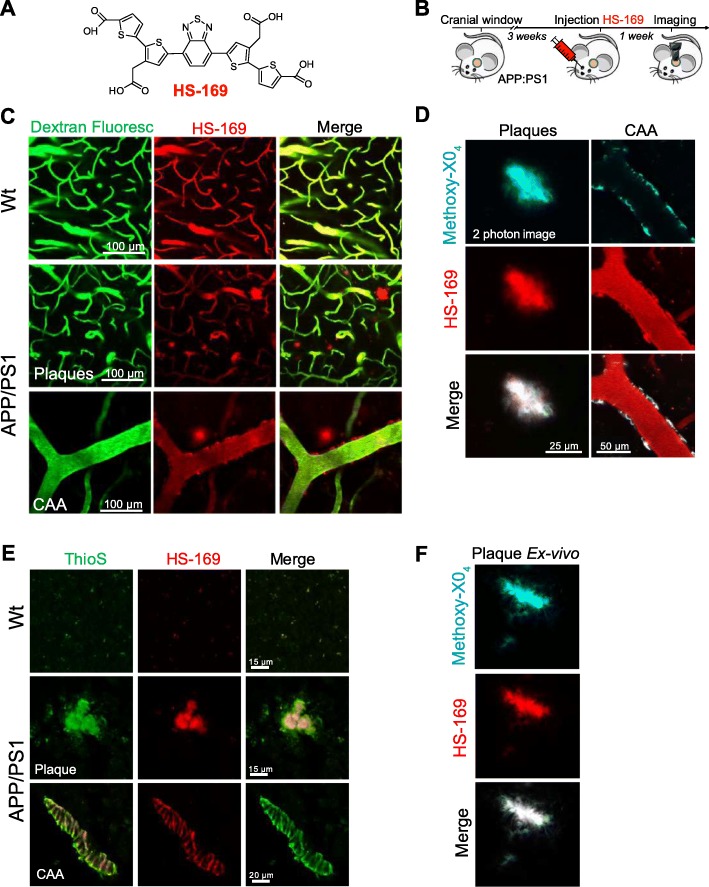
HS-169 binds to amyloid plaques and CAA in the APP:PS1 Tg mouse and can be detected with multiphoton microscopy*.* **a**. HS-169 molecule. **b**. Experimental procedure to characterize HS-169 in the mouse brain in vivo*.* APP:PS1 Tg mice and Wt littermates with a cranial window already implanted were injected with HS-169 via retro-orbital and subjected to intravital multiphoton microscopy. **c**. Representative in vivo multiphoton microscopy images of HS-169 in Wt (top) and APP:PS1 Tg mouse (middle and bottom). Pictures show Dextran Fluorescein (green), amyloid plaques and CAA labelled with HS-169 (red) and merge of both channels. Scale bar represents 100 μm and applies to all pictures. *n* = 3 Wt and 10 APP:PS1 Tg mice. **d.** In vivo validation of HS-169 labelling amyloid core plaques. HS-169 and Methoxy-X04 where co-injected in the same APP:PS1 Tg mouse. Colocalization of both dyes is shown. Representative of *n* = 3 APP:PS1 Tg mice. **e.** Post-mortem validation of HS-169 labelling amyloid beta pathology in the mouse brain. ThioS staining was used to probe colocalization with amyloid plaques and CAA in the APP:PS1 Tg mice (bottom) and compared to Wt littermates (top). HS-169 was injected via retro-orbital and the mice were euthanized 24 h later. Brains were sliced in a cryostat and incubated with 0.005% ThioS in EtOH. Colocalization of both dyes can be appreciated. **f**. Co-staining of Methoxy-X04 and HS-169 ex vivo in the APP:PS1 tissue to confirm the colocalization observed in vivo

**Fig. 4:**
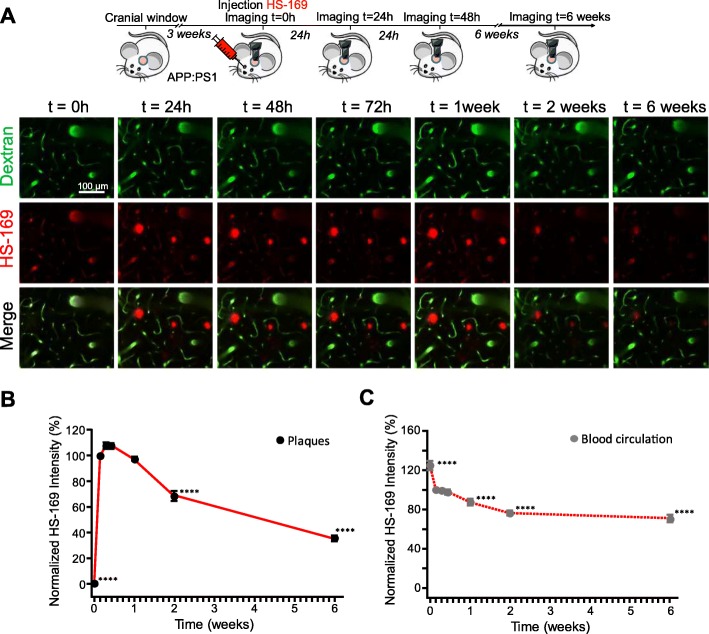
Optimal visualization window for HS-169 with multiphoton microscopy in the in vivo mouse brain. **a**. Top panel, experimental procedure to characterize HS-169 in the mouse brain in vivo*.* APP:PS1 Tg mice were retro-orbitally injected with HS-169 and longitudinally imaged with multiphoton microscopy for 6 weeks. Bottom panel, pictures show different time points after injection of HS-169 (red). t = 0 represents imaging a few minutes after injection. Dextran Fluorescein 70,000 Da MW (green) was systematically injected to create a fluorescent angiogram. Scale bar represents 100 μm and applies to all pictures. **b.** Quantitative analysis of HS-164 fluorescence intensity. Normalized to t = 24 h. Optimal imaging time was determined at 24 h after HS-169 injection in the APP:PS1 Tg mouse. *n* = 96 plaques from 2 mice. **c.** Quantitative analysis of fluorescence intensity HS-169 in the blood circulation. Normalized to t = 24 h. *n* = 75 vessels from mice

**Fig. 5:**
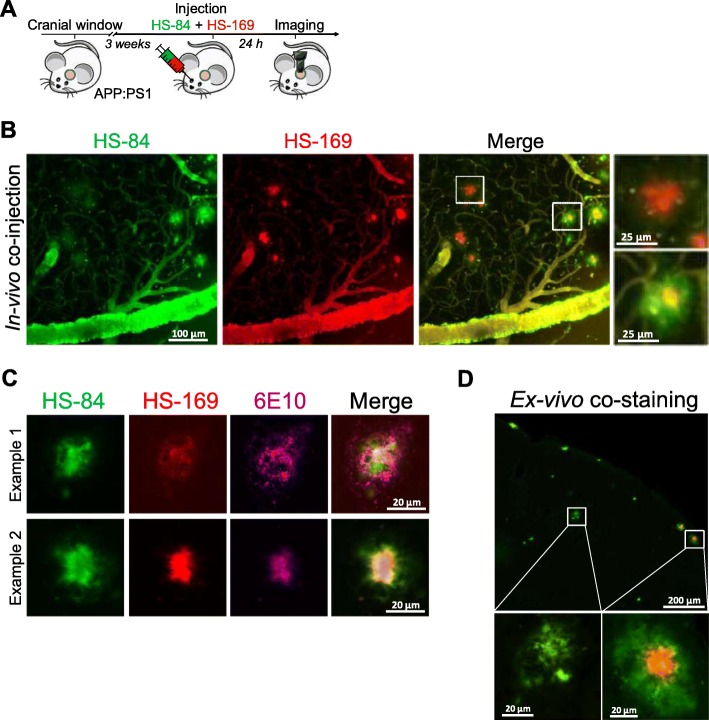
Co-injection of HS-84 and HS-169 help discriminating Aβ plaque subtypes in vivo in the APP:PS1 mouse living brain. **a**. Experimental procedure to characterize HS-169 and HS-84 in the mouse brain in vivo*.* APP:PS1 Tg mouse carrying a cranial window was simultaneously injected with HS-169 and HS-84 and subjected to multiphoton microscopy 24 h later. **b**. Representative in vivo multiphoton microscopy images of HS-84 (green) and HS-169 (red) in the same brain area in an APP:PS1 Tg mouse. Note heterogeneity among plaques. **c**. Examples of different amyloid beta plaques among the mouse brain. Immunohistochemistry with anti-abeta antibody (6E10) was carried out, followed by co-staining with HS-84 and HS-169 0.005% (w/v). Representative images from *n* = 5 mice. **d**. Ex-vivo co-staining of APP:PS1 Tg mouse tissue with HS-84 and HS-169. Note that within the same field of view, heterogeneity of amyloid β plaque can be observed

Next, we evaluated whether LCOs labelled β-amyloid pathology in vivo and whether it could be detected with multiphoton microscopy. Whereas there is a wide amount of fluorescent dyes for staining Aβ pathology ex-vivo [19], few of them can be imaged in vivo with multiphoton microscopy, with most of them emitting in the blue or green regions of the spectrum. The most standardised dye to image Aβ core plaques and CAA with multiphoton microscopy is Methoxy-X04 [13], which can be excited at 800 nm. On the other hand, most of the reporters used for in vivo imaging, such as the calcium indicator GCaMP [20], are based on GFP, making it difficult to multiplex with different probes. Interestingly, HS-169 (Fig. 3a) has a redshifted emission [3], making it suitable for labelling Aβ plaques and CAA and multiplexing with popular in vivo GFP-based reporters. As a model of cerebral amyloidosis, we used APPswe:PS1dE9 (APP:PS1), which generates Aβ peptides and progressively accumulates Aβ deposits as soon as 4–6 months of age [7]. HS-169 was injected intravenously 1 day prior to the imaging session in APP:PS1 mice with a cranial window already implanted (Fig. 3b). Fluorescein dextran (70,000 Da MW) was systematically injected to create a fluorescent angiogram. Figure 3c shows representative multiphoton microscopy images of dense core plaques and CAA pathology labelled with HS-169. Both were very clearly stained with HS-169, however, diffuse plaques were not clearly stained with HS-169. Wt littermates showed the LCO in the vessels, but no amyloid-like pathology was present, as expected. Moreover, colocalization of HS-169 and Methoxy-X04 in the plaques was observed in vivo with multiphoton microscopy in the APP:PS1 mice (Fig. 3d). To confirm that HS-169 labelled amyloid pathology in vivo, post-mortem ThioS staining was performed to probe colocalization of both dyes (Fig. 3e). In addition, LCO HS-169 colocalized with Methoxy-X04 after ex-vivo staining, confirming the results obtaining in vivo (Fig. 3f). To determine the optimal time of imaging amyloid pathology after HS-164 injection, we performed longitudinal multiphoton imaging of the same volumes for 6 consecutive weeks. APP:PS1 Tg mice with a previously implanted cranial window were injected with 150 nmol HS-169 and imaged at t = 0 (right after injection), and subsequent time points (Fig. 4a). The optimal imaging time was determined to be 24-72 h after HS-169 injection in the APP:PS1 Tg mouse (Fig. 4b). HS-169 also remained in the circulation for this period (Fig. 4c). LCO HS-84 was also injected in the APP:PS1 to label Aβ plaques and CAA and imaged with multiphoton microscopy (Additional file 3: Figure S3). We determined that HS-84 also labelled both Aβ plaques and CAA (Additional file 3: Figure S3A). Ex-vivo colocalization of HS-84 with Thiazine Red confirmed the HS-84 staining in vivo. Interestingly, when both LCOs (HS-84 and HS-169) were simultaneously injected into an APP:PS1 mouse, they did not completely colocalize. HS-169 seemed to label dense core plaques more vividly, whereas HS-84 labelled diffuse plaques and CAA more intensively (Fig. 5). Thus, this labelling with the two different LCOs showed heterogeneity among plaques (insets in Fig. 5). This heterogeneity was also observed ex-vivo, as shown in APP:PS1 tissue sections after immunohistochemistry with 6E10 antibody, followed by co-staining with both LCOs used in the same concentration and equivalent washing times. Two different examples are shown in Fig. 5c. Surprisingly, 6E10 staining also differed between plaques. A different example is shown in Fig. 5d, where at least two different types of Aβ plaques can be observed in the same area from the same mouse. Note that HS-169 staining (red) is more prominent in the core of the Aβ plaque, confirming our results in vivo.

## Discussion

While many dyes have been shown to stain protein preparations of fibrillary tau [21], few have been successfully used in vivo with multiphoton microscopy. Previous studies have shown that the Congo Red derivative, Methoxy-X04, delivered intravenously, rapidly detects NFTs in a Tg mouse model of tauopathy [22]. However, high concentrations of the dye were used, and the dye was not detectable in the brain within 24 h. In this study we show that HS-84 is a suitable probe for longitudinal in vivo detection of NFTs using multiphoton microscopy in the Tg mouse brain. HS-84 probe penetrates the BBB in vivo and binds to fibrillary tau. Importantly, it does not fade within hours, making longitudinal imaging possible, unlike other dyes such as FSB (1-Fluoro-2, 5-bis (3-carboxy-4-hydroxystyryl) benzene) [23]. One intravenous dose of the LCO was enough to longitudinally image the NFTs for up to 6 weeks. Contrary to HS-84, the HS-169 probe did not bind to NFTs in vivo. This observation could be explained by a weaker binding of HS-169 to the NFTs (as shown by the ex vivo data), although we cannot discard other possibilities such as limited ability of HS-169 to cross the BBB in the rTg4510 Tg mouse model (unlike the APP:PS1 mouse model) or to cross cell membranes in vivo. Additionally, we found that both HS-84 and HS-169 enable imaging of Aβ aggregates in vivo with multiphoton microscopy. One advantage of HS-169 over existing amyloid-binding dyes for in vivo imaging with multiphoton microscopy is that HS-169 has its peak of emission fluorescence around 650 nm (red channel) [3], making it a suitable tool for multiplexing in vivo with other common reporters that have their emission peak at lower wavelengths (blue and green channels). LCOs are unique molecules regarding their optical properties, since they display different emission spectra when binding to different amyloid types. Along these lines, ex vivo two photon imaging of mouse brains after systemic administration of heptamer formyl thiophene acetic acid (hFTAA) [2], a different LCO, revealed a distinct shift in the emission spectra when bound to Aβ plaques, CAA or NFTs [24]. In addition, in a similar manner to hFTAA [24], both in vivo and ex vivo data show that HS-84 tends to label diffuse plaques, whereas HS-169 labels mostly core plaques. Interestingly, here we showed that co-injection of HS-84 and HS-169 demonstrated heterogeneity among Aβ plaques in the same area of the mouse brain, pointing at different conformations of Aβ in the living brain. This fact could be explained by a diverse in vivo labelling depending on the polymorphism of the plaque. These results were also confirmed ex vivo after co-staining of the APP:PS1 sections with both LCOs. This concept has been previously described in the human brain, where amyloid polymorphisms were shown in different etiological subtypes of AD [25]. Taken together, these data suggest that LCOs HS-84 and HS-169 could be labeling different conformations of Aβ, however, we cannot exclude the possibility that the differences observed in vivo are due to different pharmacokinetics, different imaging efficiencies or a distinct manner of crossing BBB. Further research will be necessary to understand the different amyloid polymorphisms in the mouse brain.

LCOs have been proposed to bind to different disease-associated protein aggregates in animal models of several neurodegenerative diseases, and in post-mortem human brain tissue [2]. Insoluble hyperphosphorylated filamentous tau forms NFTs in AD, and they are present in other diseases known as tauopathies, which includes chronic traumatic encephalopathy, progressive supranuclear palsy, corticobasal degeneration or frontotemporal dementia and parkinsonism linked to chromosome 17 [26, 27]. In the human brain, amyloids have been linked to several pathologies besides AD, including CAA [28], Diabetes mellitus type 2 [29], or Parkinson’s disease among others [30]. By using intravenous injection of LCOs (as opposed to topical application) in mouse models combined with multiphoton microscopy, it is possible to follow the same areas in the living brain and to monitor the dynamics of the NFTs and amyloid pathology with time. Many questions remain open with regard to the mechanism of these diseases. Do NFTs induce cell death? Do other cell types surrounding the neurons bearing NFTs, such as astrocytes, undergo any modification or cell death? Or on the contrary are the NFTs benign? Further research is necessary to address the pathological mechanisms underlying the pathology of AD, as well as other tauopathies and cerebral amyloidosis diseases. Therefore, we foresee a wide spectrum of in vivo applications for the LCOs, in order to identify the molecular pathogenesis underlying different neurodegenerative diseases as well as the mechanism of treatment with novel developed drugs or immunotherapies.

## Supplementary information

**Additional file 1: Figure S1** HS-84 and Alz50 colocalize in the rTg4510 mouse brain**.** HS-84 was injected intravenously and mice were euthanized 1 week later. Immunohistochemistry with anti-tau antibodies (Alz50) was carried out to confirm colocalization of HS-84 with NFTs in the rTg4510 mouse brain. **A.** Representative fluorescence images of HS-84 (green) and Alz50 (red) in the rTg4510 Tg mice (bottom) and compared to Wt littermates (top).

**Additional file 2: Figure S2.** HS-169 cannot label NFTs in vivo or cannot be observed under multiphoton microscopy. **A.** Experimental procedure to characterize HS-169 in the mouse brain in vivo*.* rTg4510 Tg mouse carrying a cranial window was first injected with HS-169 and subjected to multiphoton microscopy. 3 weeks later, it was injected with HS-84 and the exact same brain areas were imaged. **B.** Representative in vivo multiphoton microscopy images of HS-169 (red) and HS-84 (green) in the same brain area in a rTg4510 Tg mouse. Note that HS-169 cannot label NFTs or it cannot be detected with multiphoton microscopy. Representative of *n* = 3 mice. **C.** Ex-vivo staining of APP:PS1-rTg4510 Tg mouse tissue with HS-84 or HS-169 independently. Note that both LCOs bound easier to amyloid beta plaques (intense fluorescence) than to NTFs (weak fluorescence). HS-169 needed more laser power and gain than HS-84 to observe NTFs when stained and imaged under the same controlled conditions.

**Additional file 3: Figure S3.** Related to Fig. 3. HS-84 selectively labels amyloid plaques and CAA in the APP:PS1 Tg mouse and can be detected with multiphoton microscopy*.* **A.** Experimental procedure to characterize HS-84 in the mouse brain in vivo*.* A cranial window was implanted in APP/PS1 Tg mice and Wt littermates. Three weeks later, they were injected with HS-84 via retro-orbital and subjected to intravital multiphoton microscopy. **B**. Representative in vivo multiphoton microscopy images of HS-84 in Wt (top) and APP:PS1 Tg mouse (middle and bottom). Pictures show amyloid plaques and CAA labelled with HS-84 (green), Dextran Texas Red (red), and merge of both channels. Scale bar represents 100 μm and applies to all pictures. *n* = 2 Wt and 2 APP:PS1 Tg mice. **C.** Post-mortem validation of HS-84 labelling amyloid pathology in the mouse brain. HS-169 was retro-orbitally injected and the mice were euthanized 24 h later. Brains were sliced in a cryostat. Thiazine Red staining was used to probe colocalization with HS-84 in amyloid plaques and CAA in the APP:PS1 Tg mice (bottom) and compared to Wt littermates (top).

## Data Availability

All data generated or analyzed during this study are included in this published article and its supplementary information files.

## Abbreviations

AD: Alzheimer’s disease
Aβ: Amyloid beta
BBB: Blood brain barrier
CAA: Cerebral amyloid angiopathy
LCO: Luminescent conjugated oligothiophene
NFTs: Neurofibrillary tangles
