# Supplementary figures and images for: In vivo detection of tau fibrils and amyloid β aggregates with luminescent conjugated oligothiophenes and multiphoton microscopy
Source: Acta Neuropathol Commun. 2019 Nov 8;7:171. doi: 10.1186/s40478-019-0832-1 (PMC6839235; doi:10.1186/s40478-019-0832-1)

Additional file 1: Figure S1

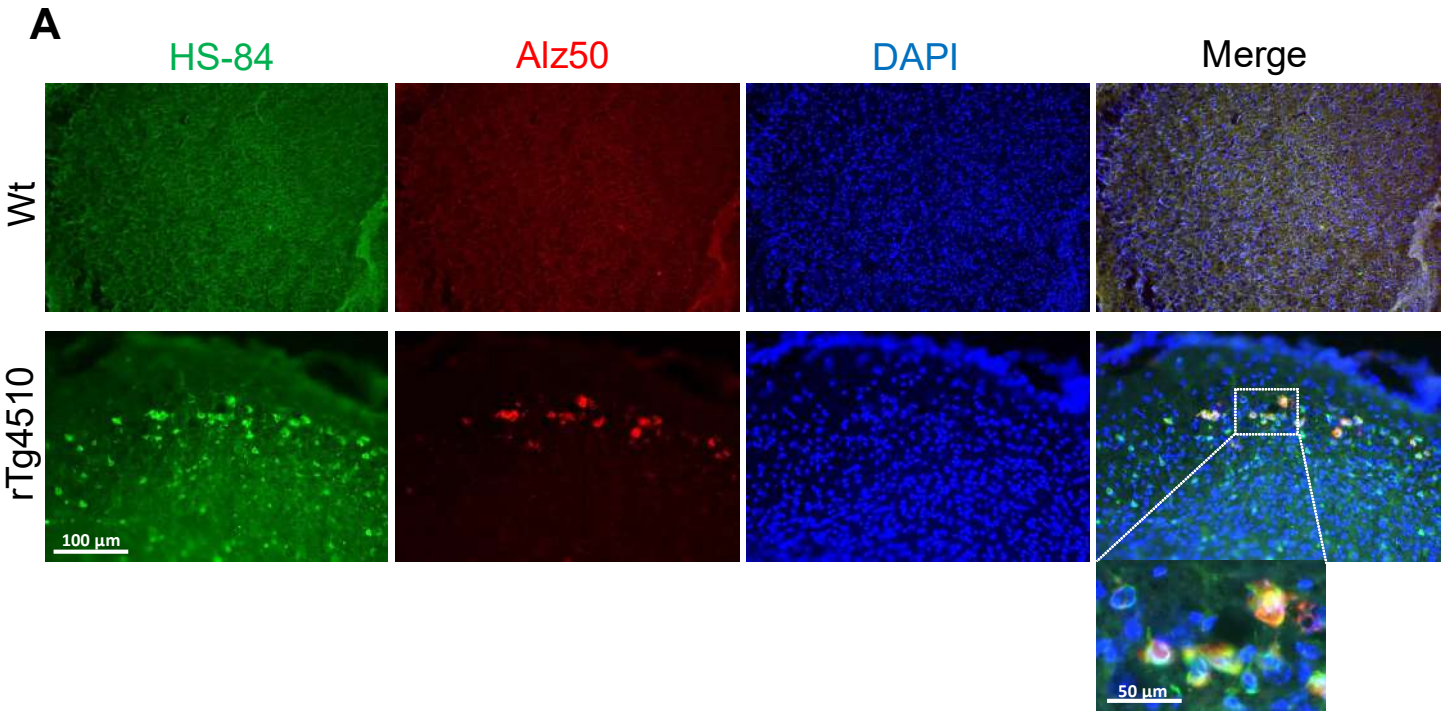

Supplement: Supplementary file 1 — Additional file 1: Figure S1 HS-84 and Alz50 colocalize in the rTg4510 mouse brain. HS-84 was injected intravenously and mice were euthanized 1 week later. Immunohistochemistry with anti-tau antibodies (Alz50) was carried out to confirm colocalization of HS-84 with NFTs in the rTg4510 mouse brain. A. Representative fluorescence images of HS-84 (green) and Alz50 (red) in the rTg4510 Tg mice (bottom) and compared to Wt littermates (top). [file 40478_2019_832_MOESM1_ESM.pdf]

# Additional file 2: Figure S2

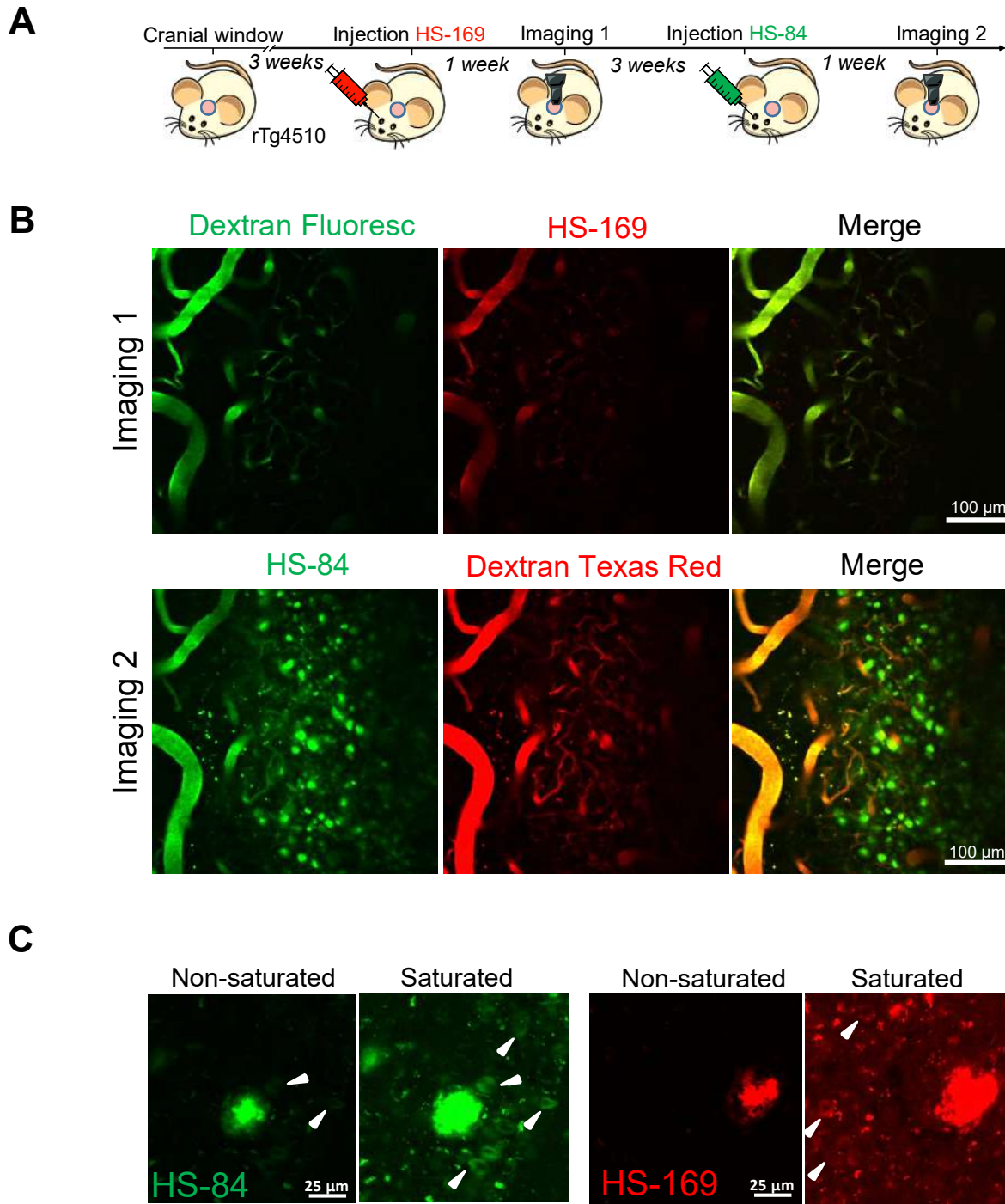

Supplement: Supplementary file 2 — Additional file 2: Figure S2. HS-169 cannot label NFTs in vivo or cannot be observed under multiphoton microscopy. A. Experimental procedure to characterize HS-169 in the mouse brain in vivo. rTg4510 Tg mouse carrying a cranial window was first injected with HS-169 and subjected to multiphoton microscopy. 3 weeks later, it was injected with HS-84 and the exact same brain areas were imaged. B. Representative in vivo multiphoton microscopy images of HS-169 (red) and HS-84 (green) in the same brain area in a rTg4510 Tg mouse. Note that HS-169 cannot label NFTs or it cannot be detected with multiphoton microscopy. Representative of n = 3 mice. C. Ex-vivo staining of APP:PS1-rTg4510 Tg mouse tissue with HS-84 or HS-169 independently. Note that both LCOs bound easier to amyloid beta plaques (intense fluorescence) than to NTFs (weak fluorescence). HS-169 needed more laser power and gain than HS-84 to observe NTFs when stained and imaged under the same controlled conditions. [file 40478_2019_832_MOESM2_ESM.pdf]

# Additional file 3: Figure S3

**A**

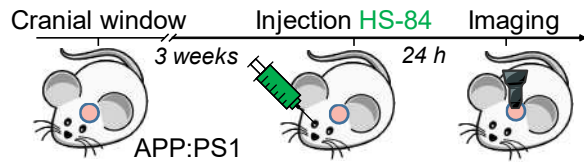

**B**

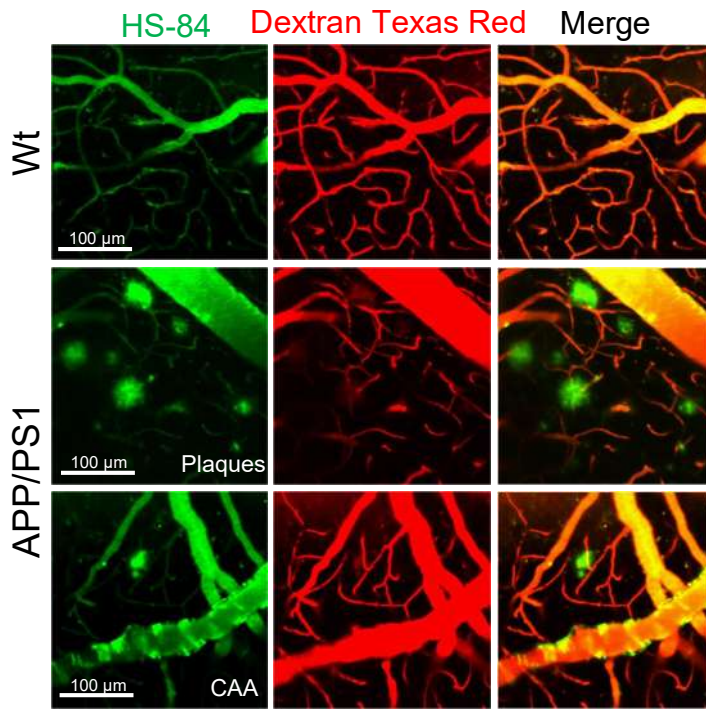

**C**

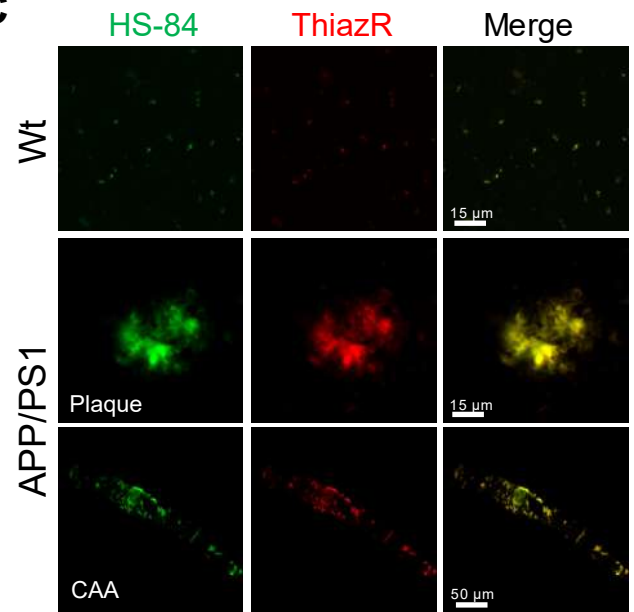

Supplement: Supplementary file 3 — Additional file 3: Figure S3. Related to Fig. 3. HS-84 selectively labels amyloid plaques and CAA in the APP:PS1 Tg mouse and can be detected with multiphoton microscopy. A. Experimental procedure to characterize HS-84 in the mouse brain in vivo. A cranial window was implanted in APP/PS1 Tg mice and Wt littermates. Three weeks later, they were injected with HS-84 via retro-orbital and subjected to intravital multiphoton microscopy. B. Representative in vivo multiphoton microscopy images of HS-84 in Wt (top) and APP:PS1 Tg mouse (middle and bottom). Pictures show amyloid plaques and CAA labelled with HS-84 (green), Dextran Texas Red (red), and merge of both channels. Scale bar represents 100 μm and applies to all pictures. n = 2 Wt and 2 APP:PS1 Tg mice. C. Post-mortem validation of HS-84 labelling amyloid pathology in the mouse brain. HS-169 was retro-orbitally injected and the mice were euthanized 24 h later. Brains were sliced in a cryostat. Thiazine Red staining was used to probe colocalization with HS-84 in amyloid plaques and CAA in the APP:PS1 Tg mice (bottom) and compared to Wt littermates (top). [file 40478_2019_832_MOESM3_ESM.pdf]
